# QA^2^FDet: Quality-Aware Adaptive Alignment Fusion Network for UAV RGBT Tiny Pedestrian Detection

**DOI:** 10.3390/s26113443

**Published:** 2026-05-29

**Authors:** Yifang Tan, Lijun Yuan, Chuanjiang Xie, Chao Zhou, Xin Li, Xinyu Zhu

**Affiliations:** College of Aviation Electronics and Electrical Engineering, Civil Aviation Flight University of China, Guanghan 618307, China; tanyf@cafuc.edu.cn (Y.T.); yuanlijun33320@163.com (L.Y.); cj_x_edu@163.com (C.X.); lixin@cafuc.edu.cn (X.L.); zhuxinyu339@163.com (X.Z.)

**Keywords:** aerial images, tiny person detection, RGBT fusion, feature alignment, quality prior

## Abstract

Visible–thermal tiny pedestrian detection in UAV aerial images is crucial for online decision-making in urban security and disaster response. However, the extremely small scale and sparse distribution of pedestrians cause discriminative cues to be submerged by dominant low-frequency background and contextual redundancy during feature learning. Meanwhile, cross-modal spatial misalignment and spatially varying modality reliability hinder stable fine-grained correspondence, thereby degrading fusion quality. To address these issues, QA^2^FDet is proposed as a quality-aware adaptive alignment fusion network comprising three modules: spectrum-spatial decoupled enhancement module (SDE), cross-modal correspondence mining module (CCM), and prior-informed gated fusion (PGF). SDE leverages the discrete cosine transform to disentangle redundant low-frequency background information, while deep semantic gating propagates high signal-to-noise ratio details into shallow representations to enhance subtle cues of tiny pedestrians and suppress high-frequency noise. To establish fine-grained neighborhood correspondences under slight spatial offsets, thermal-guided local asymmetric cross-attention is designed in CCM. Finally, region-level quality and modality discrepancy are jointly modeled for adaptive cross-modal fusion in PGF. Extensive experiments on multiple UAV-based RGBT detection benchmarks demonstrate that QA^2^FDet achieves state-of-the-art performance and exhibits strong robustness in challenging aerial scenes.

## 1. Introduction

The rapid development of the low-altitude economy and intelligent emergency response systems is driving UAV vision from offline analysis toward online decision-making, where aerial pedestrian detection is increasingly regarded as a critical enabling capability for urban security, disaster rescue, and infrastructure inspection [1,2,3]. Compared with ground-view settings, pedestrians in aerial images are typically characterized by extremely small scales and sparse distributions, and their detection is continually degraded by complex background textures, nadir-view geometric distortions, and motion blur [4]. Under nighttime operation, backlighting, smoke occlusion, or thermally cluttered environments, the instability of visible cues further constrains the performance ceiling of single-modality detectors; consequently, visible–thermal (RGBT) fusion has been widely adopted to improve robustness for UAV-based tiny pedestrian detection [5,6]. Nevertheless, existing RGBT fusion approaches remain challenged by the intertwined effects of cross-modal geometric misalignment, time-varying modality quality, and weak tiny-object representations, where even slight spatial offsets can obscure discriminative cues [7]. Meanwhile, modality reliability is often spatially and temporally non-uniform, such that stable cross-modal correspondences are difficult to establish and region-wise adaptive, preferential fusion is difficult to achieve, ultimately leading to systematic coexistence of missed detections and false alarms [8,9]. Accordingly, RGBT tiny pedestrian detection on UAV platforms is still an active and challenging research problem.

In practical UAV deployment, onboard perception is constrained by stringent platform-level hardware limitations, including payload capacity, power budget, memory footprint, computational throughput, and thermal dissipation [10]. These limitations hinder the deployment of heavyweight detectors and computationally intensive global multimodal fusion modules, especially when real-time inference is required for online decision-making [11]. RGBT perception further increases system complexity, as paired visible–thermal sensors require accurate calibration, temporal synchronization, and concurrent multimodal data acquisition and transmission [12]. Consequently, a UAV-oriented RGBT detector is expected to remain robust to tiny-object scales, cross-modal misregistration, and spatially varying modality reliability, while maintaining a favorable trade-off among detection accuracy, parameter size, computational cost, and inference speed [13]. These platform-level hardware constraints further emphasize the need for efficient and reliability-guided RGBT fusion strategies tailored to UAV-based tiny pedestrian detection.

Aerial object detection remains intrinsically challenging due to nadir-dominant viewing geometry, arbitrary orientations, and large-scale variation. Ding et al. [14] summarized key factors, including scale changes, oriented instances, and cluttered-scene interference on the DOTA benchmark, providing a unified context for method evaluation; however, the analysis is primarily oriented toward generic aerial objects and does not explicitly address the more severe weak object representation and cross-modal coupling disturbances encountered in UAV-based tiny pedestrian detection. Oriented R-CNN [15] improves efficiency by generating rotated proposals in a streamlined manner. Li et al. [16] strengthen rotation and shape modeling via the point-set geometric representation of Oriented RepPoints, and Yu et al. [17] further reduce annotation cost by exploring point-level supervision for oriented detection. Nevertheless, these methods are largely centered on geometric formulation and annotation paradigms, while explicit suppression of low-frequency background dominance and redundant contextual interference in tiny pedestrian feature learning has been insufficiently investigated. For high-resolution small objects detection, QueryDet [18] accelerates inference and enhances accuracy through cascaded sparse queries, yet the improvement is mainly achieved by alleviating computational overhead and query design, and the progressive submergence of tiny-object cues by low-frequency background components and local noise across multi-level feature refinement is not fundamentally avoided. In addition, Miri Rekavandi et al. [19] and Hua et al. [20] review advances in multi-scale modeling, contextual reasoning, and attention mechanisms from transformer-based and aerial small-object perspectives and consistently identify the long-standing bottlenecks of sparse discriminative cues and strong background interference. Overall, prevailing approaches still predominantly rely on spatial-domain stacking for enhancement, whereas controllable decoupling between background redundancy and fine-grained object details remains limited.

To improve robustness under low illumination, backlighting, and occlusion, RGBT pedestrian detection has been widely investigated with an emphasis on fusion, alignment, and false-positive suppression. Song et al. [21] and Lu et al. [22] systematically review RGBT task taxonomies and fusion techniques, respectively, providing a high-level backdrop for multimodal perception. From the detection perspective, however, cross-modal misregistration, time-varying modality quality, and task-driven adaptive fusion remain open challenges. To alleviate false alarms induced by fusion noise, TFDet [23] suppresses task-irrelevant noise propagation via object-aware fusion, but the imposed constraints are largely coarse-grained. When pedestrians are extremely small and highly sensitive to pixel-level shifts, critical regions may still suffer from mismatched fusion. MS-DETR [24] introduces a DETR-style decoder with loosely coupled sampling to improve tolerance to mild misalignment and further mitigates modality imbalance through modality-balancing optimization. Nevertheless, for tiny and sparse objects, cross-modal correspondences are more easily perturbed by background textures, and the joint modeling of local fine-grained correspondence and weak object representation remains insufficient. For reliability modeling, CMPD [25] leverages Dempster–Shafer evidence theory to assign confidence for fusion guidance, and Li et al. [26] further enhance stability by combining cross-modal homogeneity reinforcement with confidence-aware fusion. However, reliability is often estimated globally or at a coarse spatial resolution, making it difficult to yield region-level preference cues that remain sensitive to tiny objects in UAV scenarios where smoke occlusion, thermal distractors, and misalignment can co-occur. To address misregistration, Zhang et al. [27] explicitly learn spatial and modality alignment; DeformCAT [28] employs deformable cross-attention for weakly aligned RGBT pedestrians; DAMSDet [29] handles time-varying complementarity and misalignment via dynamic query selection and adaptive fusion; and Hou et al. [30] formulate fusion logic from similarity and complementarity. Overall, alignment and reliability selection are still frequently treated in isolation: alignment modules are seldom constrained by region-level quality priors, while reliability weighting cannot ensure the fine-grained spatial correspondence that is most critical for tiny objects. Meanwhile, DLA-Deformable DETR [31] improves sparse sampling and alignment via deformable attention; Swin Transformer [32] is introduced as a hierarchical vision Transformer backbone; DAB-DETR [33], DN-DETR [34], and DINO [35] advance DETR variants through dynamic anchor queries, denoising training, and improved denoising anchors; ConvNeXt [36] and ConvNeXt V2 [37] exemplify modern CNN-based representation backbones; and InternImage [38] pushes real-time end-to-end detection toward an NMS-free paradigm. These developments primarily strengthen generic representation and inference schemes but are not explicitly tailored to UAV RGBT tiny pedestrian detection, where fine details are submerged by low-frequency background components, and robust fusion requires fine-grained correspondence together with region-level reliability prioritization under weak misalignment. In the frequency domain, FcaNet [39] and AIS-FCANet [40] show that frequency cues can be incorporated into channel-attention modeling to enhance structural representation, supporting the utility of frequency information for structure encoding; however, they are not task-specifically designed for the coupled detection challenges posed by multimodal misalignment and time-varying reliability.

Recent RGBT salient object detection methods have provided relevant insights into modality-aware interaction and adaptive fusion. For instance, Zhang et al. proposed an asymmetric light-aware progressive decoding network for RGBT salient object detection, where asymmetric cross-modal interaction, light-aware feature selection, and progressive decoding are employed to suppress modality interference and refine salient regions [41]. Luo et al. developed a Transformer-based cross-modality interaction guidance network (CIGNet) to guide complementary RGBT feature interaction [42], while Zhao et al. introduced a wavelet-driven multi-band feature fusion strategy to integrate low- and high-frequency cross-modal cues for robust saliency prediction [43]. However, they are mainly designed for dense pixel-level saliency prediction, whereas UAV-based RGBT tiny pedestrian detection requires box-level localization of sparse and extremely small targets under scale degradation, cross-modal misregistration, and spatially varying modality reliability.

Based on the analysis of the aforementioned methods, current UAV-based RGBT fusion methods for tiny pedestrian detection have two key bottlenecks that limit their effectiveness:

Issue 1: Tiny-object cues are readily overwhelmed by low-frequency background and contextual redundancy, yielding insufficient discriminative features. Consequently, during iterative learning, detection heads are biased toward background structures or local noise rather than tiny pedestrians.

Issue 2: Tiny objects are highly sensitive to cross-modal misregistration, but fusion is often performed without fine-grained reliability guidance. On UAV platforms, parallax, calibration errors, and temporal asynchrony can induce spatial misalignment between visible and thermal modalities. For extremely small objects, even minor shifts can severely impair cross-modal complementarity. When fusion is controlled only by coarse-grained or global weights, mismatched fusion at critical regions is likely, degrading sensitivity to tiny pedestrians.

To tackle these issues, we propose QA^2^FDet, a quality-aware adaptive alignment and fusion network for UAV RGBT tiny pedestrian detection. Given paired visible and thermal images, a dual-stream backbone extracts initial multi-scale modality-specific features, which are then processed by the spectrum–spatial decoupled enhancement (SDE) module. In SDE, the block-wise discrete cosine transform (Block-DCT) spectral decoupling explicitly separates low-frequency background redundancy and extracts high signal-to-noise ratio detail maps. Different from generic frequency-attention designs, these details are selectively injected into shallow features under deep semantic gating, thereby enhancing detection-relevant tiny pedestrian cues while avoiding indiscriminate amplification of high-frequency noise. To mitigate the high sensitivity of tiny objects to cross-modal misalignment, the cross-modal correspondence mining (CCM) module performs thermal-guided asymmetric local cross-attention, where thermal tokens serve as spatial anchors and visible candidates are searched within neighboring windows to establish fine-grained local correspondences. Spatially varying modality reliability is further estimated by the quality prior estimator (QPE), which derives fine-grained quality-prior maps from modality-specific classification and regression responses, providing detection-quality-supervised region-level reliability cues for subsequent prior-informed fusion after local correspondence mining. Finally, the prior-informed gated fusion (PGF) module jointly models quality priors and modality-difference cues to generate bidirectional adaptive gates, enabling degraded or redundant modality responses to be suppressed while complementary object-sensitive cues are enhanced. The fused features are then forwarded to the detection head for final prediction. Overall, QA^2^FDet establishes a quality-aware feature refinement mechanism for UAV RGBT tiny pedestrian detection, in which modality-specific responses are progressively purified, locally calibrated, and reliability-adapted toward detection-sensitive representations. This integrated design enhances the detector’s ability to preserve tiny pedestrian cues under background clutter, weak cross-modal misregistration, and spatially varying modality reliability.

The main contributions can be summarized as follows:

- A spectrum–spatial decoupled feature enhancement strategy is developed to suppress dominant low-frequency background responses and selectively introduce high signal-to-noise detail cues into shallow representations, improving the discriminability of tiny pedestrian features.
- Thermal responses are exploited as spatial anchors to mine local visible candidates within neighboring regions, enabling fine-grained cross-modal correspondence modeling under slight visible–thermal spatial offsets.
- A prior-informed bidirectional gated fusion strategy is proposed to jointly exploit region-level reliability and modality-discrepancy cues, adaptively suppressing degraded or redundant responses while enhancing complementary object-sensitive information.
- Extensive experiments on three UAV-oriented RGBT benchmarks demonstrate the superior detection accuracy, cross-modal resilience, and deployment-oriented computational efficiency of the proposed method in challenging aerial scenarios.

## 2. Methods

### 2.1. Framework Overview

**Figure 1:**
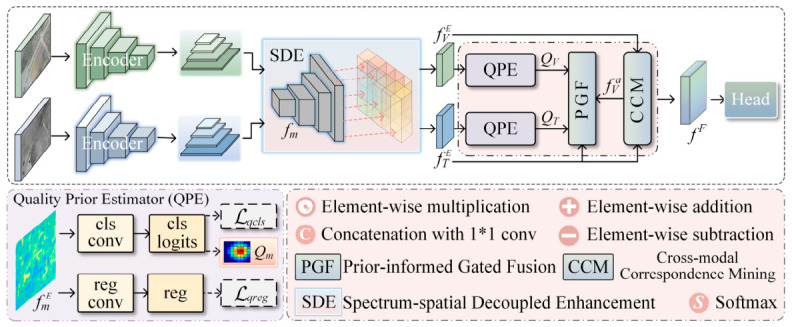
The overall framework of QA^2^FDet. The notation panel at the bottom provides a unified legend for the schematic figures, including element-wise operations, concatenation, Softmax, and the abbreviations of the main modules.

The overall framework of QA^2^FDet is illustrated in Figure 1. A visible image $I_{V}\in R^{H\times W\times 3}$ and a thermal image $I_{T}\in R^{H\times W\times 1}$ are processed by a dual-stream backbone, from which multi-scale features ${\left\{f_{m}^{l}\right\}}_{l=1}^{5}\;\left(m\in \left\{V,T\right\}\right)$ are extracted for each modality. These features are then fed into SDE, where Block-DCT is applied to explicitly strip low-frequency redundancy and produce detail maps; under gated deep semantic priors, high signal-to-noise ratio details are injected into shallow features, yielding enhanced representations $f_{m}^{E}$. The enhanced features are subsequently processed by QPE, in which auxiliary classification and regression supervision are imposed to generate spatially fine-grained quality-prior maps ${Q_{V},Q}_{T}\in R^{H\times W}$ for quantifying region-wise modality reliability. To address the high sensitivity of tiny objects to cross-modal misregistration, CCM leverages thermal-guided asymmetric cross-attention to mine fine-grained cross-modal correspondences within local neighborhoods, thereby aligning the visible features to $f_{V}^{a}$. Finally, PGF jointly models the quality priors and modality-discrepancy weights to realize adaptive gated fusion, and the fused features are forwarded to the detection head to produce the final predictions.

### 2.2. Spectrum-Spatial Decoupled Enhancement Module

**Figure 2:**
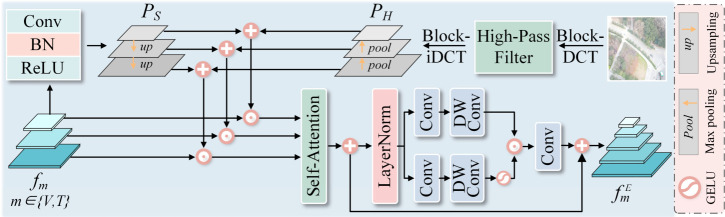
The framework of the spectrum-spatial decoupled enhancement module (SDE).

During iterative optimization, tiny-object features are prone to being submerged by large-area low-frequency backgrounds and redundant contextual information. To alleviate this issue, SDE is proposed, as shown in Figure 2. SDE is composed of two complementary streams. In the spectral decoupling stream, a block-wise discrete cosine transform (Block-DCT) is applied to explicitly separate high-frequency object details from low-frequency background redundancy. In parallel, the semantic guidance stream leverages deep features as spatial priors; detail injection is gated to suppress noise and prevent the propagation of unreliable cues. The enhanced representations are subsequently refined using lightweight attention and multi-branch depthwise convolutions.

For an input visible or thermal image $I_{m}$, Block-DCT is performed within each 8 × 8 frequency block to conduct spectral analysis while preserving local spatial topology. A high-pass spectral mask $M_{H}$ is further designed as a filtering operator:

(1) $$\rho \left(u,v\right)=\frac{{∥\left[u,v\right]∥}_{2}}{{∥\left[7,7\right]∥}_{2}}$$

(2) $$M_{H}=\sigma \left(\rho \left(u,v\right)-\gamma_{m}\right)$$

where $\left(u,v\right)\in {\left\{0,\ldots ,7\right\}}^{2}$ denotes the 2D frequency coordinates, ρ∈[0,1] is the normalized frequency radius, ${∥\cdot ∥}_{2}$ represents the Euclidean norm, σ(·) is the sigmoid function, and $\gamma_{m}\in (0,1)$ is a modality-specific learnable frequency-band threshold. With this design, low-frequency components are softly attenuated, whereas high-frequency components that encode salient object-boundary cues are preferentially preserved. The filtered spectrum is then projected back to the spatial domain via the block-wise inverse discrete cosine transform (Block-iDCT), producing the decoupled detail map $D_{m}$ in which background redundancy is effectively suppressed:

(3) $$D_{m}=D^{-1}\left(M_{H}⊙D\left(I_{m}\right)\right)$$

where D(·) and $D^{-1}\left(\cdot \right)$ denote the Block-DCT and Block-iDCT, respectively, each applied independently to every 8 × 8 block. The operator ⊙ denotes element-wise multiplication. Subsequently, a detailed feature pyramid $P_{H}^{l}$ aligned with the backbone hierarchy is constructed via max pooling:

(4) $$P_{H}^{l}=MaxPool\left(D_{m},s^{l}\right),\;l\in \left\{1,2\right\}$$

where $s^{l}$ denotes the effective stride of the l-th scale relative to the input image. Although frequency-domain enhancement preserves boundary structures, it may also amplify high-frequency environmental noise. To mitigate this effect, a semantic-guidance stream is constructed, in which high-level semantics are leveraged as spatial priors to gate denoising. Specifically, the deep feature $f_{m}^{3}$ is channel-aligned via an adapter ϕ(⋅), which consists of a convolution layer followed by batch normalization and ReLU activation, and is then upsampled by Up(·) to the current scale, yielding the semantic prior $P_{S}^{l}$:

(5) $$P_{S}^{l}=Up\left(\phi (f_{m}^{3}),s^{3-l}\right),l\in \left\{1,2\right\}$$

Subsequently, the fine-grained texture cues in $P_{H}^{l}$ and the coarse semantic priors in $P_{S}^{l}$ are fused to jointly exploit micro-level details and macro-level localization cues:

(6) $$W_{E}=\sigma (P_{S}^{l}+P_{H}^{l})$$

The learned weights are then multiplied element-wise with the original feature to obtain the enhanced intermediate feature ${\tilde{f}}_{m}$:

(7) $${\tilde{f}}_{m}=f_{m}⊙W_{E}$$

To improve the global consistency of the gated features and further refine the feature representation, a lightweight attention mechanism and multi-branch depthwise convolutions are incorporated at each scale.

(8) $$Z_{m}=LN\left({\tilde{f}}_{m}+SelfAttn\left({\tilde{f}}_{m}\right)\right)$$

where LN(·) denotes LayerNorm, and SelfAttn(·) denotes multi-head self-attention with 4 heads. The features are then processed by a parallel-branch refinement module for nonlinear transformation, resulting in the enhanced feature $f_{m}^{E}$:

(9) $$f_{m}^{E}=Z_{m}+C_{3}\left(DW\left(C_{1}Z^{m}\right)⊙GELU\left(DW\left(C_{2}Z_{m}\right)\right)\right)$$

where DW(·) denotes a 3 × 3 depthwise convolution. $C_{1}$ and $C_{2}$ are 1 × 1 convolutions used for channel expansion, whereas $C_{3}$ is a 1 × 1 convolution used to compress channels back to the original dimensionality.

### 2.3. Cross-Modal Correspondence Mining Module

**Figure 3:**
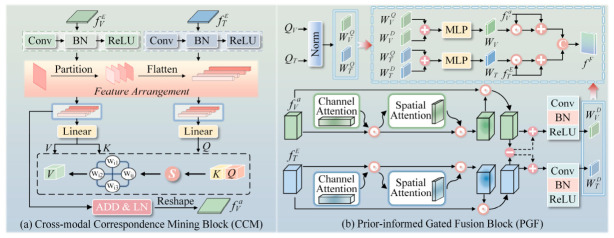
The framework of (**a**) Cross-modal correspondence mining module (CCM), and (**b**) Prior-informed gated fusion (PGF).

To explicitly model spatial correspondences between the two modalities and enable fine-grained alignment, a CCM module is designed, as illustrated in Figure 3a. Taking the high signal-to-noise ratio spectral features $f_{V}^{E}$ and $f_{T}^{E}$ produced by the SDE as inputs, CCM performs asymmetric cross-window attention to search for local matches within neighborhood windows, thereby mining cross-modal correspondences and generating more spatially aligned representations.

Considering that the thermal modality provides more reliable geometric localization for tiny objects in complex environments, a thermal-guided asymmetric alignment strategy is proposed. Specifically, $f_{V}^{E},f_{T}^{E}\in R^{H\times W\times C}$ are first passed through modality-specific adaptation layers for nonlinear mapping. The resulting feature maps are then partitioned into b × b non-overlapping windows and flattened into token sequences $X_{m}\in R^{N\times b^{2}\times C}$, where $N=HW/b^{2}$ denotes the number of windows. A d-dimensional embedding is then obtained via linear projection. Two linear projections are then applied: the thermal token sequence is projected to Query, whereas the visible neighborhood token sequence is projected to Key and Value:

(10) $$Q={Linear(X}_{T}),\;[K,V]=Linear(X_{V})$$

where $Q,K,V\in R^{N\times b^{2}\times d}$. To achieve stable window-level matching without sacrificing token-level representations, window-wise aggregation is applied to Q and K, producing $q_{i}$ and $k_{j}$:

(11) $$q_{i}=GAP\left(Q_{i}\right),k_{j}=GAP\left(K_{j}\right)$$

where $q_{i}\in R^{d}$, $k_{j}\in R^{d}$, and GAP(·) denotes global average pooling. Here, i∈{1,2,…,N} indexes the thermal query window, whereas j∈N(i)⊆{1,2,…,N} indexes the visible candidate windows. The candidate set N(i) is defined as the 3 × 3 spatial neighborhood centered at window i. The neighborhood-wise attention weights are then computed as:

(12) $$w_{ij}=\frac{exp\left(\frac{q_{i}^{⊤}k_{j}}{\sqrt{d_{k}}}\right)}{\sum_{j\in N\left(i\right)}exp\left(\frac{q_{i}^{⊤}k_{j}}{\sqrt{d_{k}}}\right)}$$

where $d_{k}$ denotes the channel dimension of k. The resulting distribution is used to weight and aggregate V across the candidate visible windows, yielding the visible residual aligned with thermal window i:

(13) $${Atten(Q,K,V)}_{i}=\sum_{j\in N(i)}w_{ij}V_{j}$$

To facilitate gradient backpropagation and stabilize the feature distribution, the attention-layer output is combined with the original visible feature sequence $X_{V}$ via a residual connection, followed by Layer Normalization LN(·):

(14) $$X_{V}^{a}=LN(X_{V}+Atten(Q,K,V))$$

Finally, the sequence features are reshaped to restore the 2D spatial structure, generating the final aligned visible feature map $f_{V}^{a}$.

### 2.4. Quality Prior Estimator

As shown in Figure 1, QPE applies a lightweight prediction head to $f_{m}^{E}$ to produce the classification and regression outputs, respectively:

(15) $$F_{m}^{cls}=g_{cls}\left(f_{m}^{E}\right),F_{m}^{reg}=g_{reg}\left(f_{m}^{E}\right)$$

where $g_{cls}(\cdot )$ and $g_{reg}(\cdot )$ are implemented as convolutional mappings. $F_{m}^{cls}$ denotes the classification logits, and $F_{m}^{reg}$ denotes the regression output, parameterized consistently with the detection head. The outputs from the classification and regression branches are then concatenated along the channel dimension and further regressed to yield the spatial quality prior map:

(16) $$Q_{m}=\sigma \left(Conv\left(Concat\left[F_{m}^{reg},F_{m}^{cls}\right]\right)\right)$$

### 2.5. Prior-Informed Gated Fusion

As illustrated in Figure 3b, to convert $\left\{{Q_{V},Q}_{T}\right\}$ into modality reliability weights that are directly applicable to gating, the quality maps of both modalities are normalized, gaining the quality weights $W_{V}^{Q}$ and $W_{T}^{Q}$:

(17) $$\begin{array}{c} W_{V}^{Q}=\frac{Q_{V}-min\left(Q_{V},Q_{T}\right)}{max\left(Q_{V},Q_{T}\right)-min\left(Q_{V},Q_{T}\right)}\\ W_{T}^{Q}=\frac{Q_{T}-min\left(Q_{V},Q_{T}\right)}{max\left(Q_{V},Q_{T}\right)-min\left(Q_{V},Q_{T}\right)} \end{array}$$

Channel and spatial attention are applied to the two modalities of features $f_{V}^{a}$ and $f_{T}^{E}$, respectively, producing the denoised representations:

(18) $${\hat{f}}_{V}=SA\left(CA\left(f_{V}^{a}\right)\right),{\hat{f}}_{T}=SA\left(CA\left(f_{T}^{E}\right)\right),$$

where CA(·) and SA(·) denote the channel-attention and spatial-attention modules, respectively. Subsequently, the modality-specific difference features $\Delta_{V}$ and $\Delta_{T}$ are constructed as follows:

(19) $$\Delta_{V}={\hat{f}}_{V}-{\hat{f}}_{T},\Delta_{T}={\hat{f}}_{T}-{\hat{f}}_{V}.$$

The difference feature $\Delta_{m}$ captures the regions in modality m that are complementary with respect to the other modality, whereas ${\hat{f}}_{m}$ provides modality-specific contextual information. The two are jointly encoded, and a difference-aware weight map is generated via ϕ(⋅):

(20) $$W_{m}^{D}=\sigma \left(\phi \left(Concat\left[{\hat{f}}_{m},\Delta_{m}\right]\right)\right)$$

After the quality weights are fused with the difference weights, the resulting fused weights are fed into a multi-layer perceptron (MLP) to yield the final gating coefficients:

(21) $$W_{m}=2\sigma \left(MLP\left(W_{m}^{Q}+W_{m}^{D}\right)\right)-1$$

where $W_{m}\in [-1,1]$ allows bidirectional modulation, enabling each modality feature to be selectively enhanced or suppressed. The two modality features are then adaptively scaled using a gated-residual mechanism, and the resulting gated features are concatenated along the channel dimension to produce the final fused representation $f^{F}$:

(22) $$f^{F}=Concat\left[f_{V}^{a}⊙{exp(W}_{V}),f_{T}^{E}⊙{exp(W}_{T})\right]$$

### 2.6. Loss Function

The training objective of QA^2^FDet is formulated as the sum of the detection head loss $L_{det}$ and the quality-prior auxiliary loss $L_{qpe}$. The former is used to supervise the final predictions derived from the fused features, whereas the latter is introduced to enforce that QPE learns a stable spatial quality map, thereby providing a reliable prior for the subsequent PGF module. The overall loss is defined as:

(23) $$L=L_{det}+L_{qpe}$$

(24) $$L_{det}=L_{reg}+L_{cls}=GIOU\left(b,b^{*}\right)+FL\left(c,c^{*}\right)$$

(25) $$L_{qpe}=L_{qreg}+L_{qcls}+L_{q}$$

where $L_{reg}$ and $L_{qreg}$ denote the bounded bounding-box regression losses for the detection head and QPE, respectively, while $L_{cls}$ and $L_{qcls}$ denote the corresponding classification losses. GIOU(·) and FL(·) denote the GIoU loss and the Focal loss, respectively. b and c correspond to the regression and classification predictions, and the superscript * denotes the ground-truth object. $L_{q}$ represents the quality-map regression loss. The regression branches are supervised by the GIoU loss [44], and the classification branches are supervised by the Focal loss [45]. $L_{qreg}$ and $L_{qcls}$ are given by:

(26) $$L_{qreg}=GIOU\left(b_{v},b^{*}\right)+GIOU\left(b_{t},b^{*}\right)$$

(27) $$L_{qcls}=FL\left(c_{v},c^{*}\right)+FL\left(c_{t},c^{*}\right)$$

where the subscripts v and t indicate the visible and thermal branches, respectively. $L_{q}$ is measured by the mean squared error (MSE) loss:

(28) $$L_{q}=MSE\left(Q_{V},Q_{V}^{*}\right)+MSE\left(Q_{T},Q_{T}^{*}\right)$$

where $Q_{V}$ and $Q_{T}$ are the quality-prior maps predicted by QPE for the visible and thermal modalities, respectively. $Q_{V}^{*}$ and $Q_{T}^{*}$ are the corresponding IoU-based target quality maps, which are generated online during training. Specifically, for each positive object region, the target quality value is assigned according to the localization quality of the modality-specific QPE regression output with respect to the matched ground-truth box, i.e., the IoU between the predicted box and the ground-truth box. Therefore, $Q_{V}^{*}$ and $Q_{T}^{*}$ are generated from the visible and thermal QPE regression outputs, respectively. For background regions, the target quality values are set to 0. In this way, $L_{q}$ explicitly supervises QPE to assign higher quality responses to reliable object regions and lower responses to unreliable or background regions.

Thus, $L_{qreg}$ provides localization supervision for the modality-specific QPE regression branches, $L_{qcls}$ provides category-level supervision for the QPE classification branches, and $L_{q}$ directly constrains the predicted quality-prior maps. Together, these three terms enable QPE to produce spatially reliable quality priors for subsequent prior-informed gated fusion.

## 3. Experiments

### 3.1. Experimental Protocol

#### 3.1.1. Datasets

Our method was evaluated on three UAV-view RGBT datasets: RGBTDronePerson [6], a drone-based tiny person dataset with 6125 paired visible–thermal images, split into 4900 for training and 1225 for testing, covering three person-centric categories (Person, Rider, and Crowd); VTUAV-det [6], a detection benchmark re-annotated from the VTUAV tracking dataset, containing 16,770 paired images in total (11,392 for training and 5378 for testing) across three categories (Vehicle, Person, and Cyclist); and VTSaR [46], a search-and-rescue-oriented aerial person detection dataset with 5333 paired images (4800 training and 533 testing) containing a single category (Person).

#### 3.1.2. Comparison Methods

The comparison multispectral object detection algorithms include: a UAV-view RGBT detector tailored for tiny pedestrians (QFDet [6]); RGBT pedestrian detection methods (DeformCAT [28], TFDet [23]); a representative DETR-based multispectral detector (MS-DETR [24]); confidence-aware fusion approaches (CMPD [25], MCHE-CAF [26]); strong general-purpose RGBT object detection baselines (C^2^Former [47], CAGTDet [48]); and representative methods targeting tiny objects and challenging illumination conditions (ICAFusion [49], TINet [50]).

#### 3.1.3. Evaluation Metrics

Object detection performance was evaluated using four overall metrics: mean Average Precision (mAP), inference speed (FPS), model size (Param., M), and computational complexity (GFLOPs). The mAP summarizes detection accuracy by integrating precision–recall performance under a specified intersection over union (IoU) criterion and is used as the primary indicator of detection quality. FPS (frames per second) measures the inference throughput of a model under a fixed testing setup, reflecting practical runtime efficiency. Param. (M) reports the number of learnable parameters (in millions) of the full detector, indicating model capacity as well as storage and deployment feasibility. GFLOPs reports the number of floating-point operations required for one forward pass of the full detector, measured in billions, and reflects the hardware-independent computational complexity of the model.

#### 3.1.4. Implementation Details

We implement our QA^2^FDet in PyTorch 1.10 using the MMDetectionV2 framework [51]. For fair comparison, all experiments involving the proposed method and reproduced baselines were conducted under a unified experimental protocol, with identical dataset splits, input resolution, data augmentation, training and evaluation procedures, and hardware configuration. All input images are resized to 640 × 512, and the aspect ratio is preserved by padding where necessary. The backbone network is ResNet-50 [52], pretrained on ImageNet [53]. Our model is trained for 12 epochs using the SGD optimizer with a momentum of 0.9, an initial learning rate of 0.005, a weight decay of 0.0001, and a batch size of 8. The learning rate is linearly warmed up for the first 500 iterations with a warm-up ratio of 1/3 and decayed by a factor of 0.1 at epochs 8 and 11. All loss terms are assigned a weight of 1.0.

To prevent overfitting, 10% of the training set is held out for validation, and the checkpoint with the best validation performance is selected as the final model. For the final models trained on RGBTDronePerson, VTUAV-det, and VTSaR, the learned thresholds converge to ($\gamma_{V},\;\gamma_{T}$) = (0.43, 0.36), (0.41, 0.34), and (0.45, 0.38), respectively. For data augmentation, only synchronized random horizontal flipping with a probability of 0.5 is applied to each visible–thermal image pair and the corresponding bounding boxes. During testing, the input size remains unchanged, the pre-NMS top-k is set to 1000, and the maximum number of detections per image is set to 100. The NMS IoU threshold is set to 0.3 for RGBTDronePerson and 0.6 for VTUAV-det and VTSaR. The confidence threshold is fixed to 0.05. All experiments are conducted on an NVIDIA GeForce RTX 5070 Ti GPU and an AMD Ryzen 7 9700X 8-Core Processor CPU.

### 3.2. Comparison with State-of-the-Art Methods

#### 3.2.1. Comparison on RGBTDronePerson

(1) Quantitative Comparison and Analysis: In Table 1, a comparison with existing multi-category RGBT detectors reveals that certain multispectral fusion methods introduce significant structural and computational complexity during the fusion stage, yet fail to achieve substantial gains for tiny objects. For instance, TINet, with a model size of 100.6 M parameters, exhibits a noticeable performance gap compared to our QA^2^FDet on this dataset, falling behind by 39.2%/41.5% in ${mAP}_{50}^{all}$/${mAP}_{50}^{tiny}$. From the efficiency perspective, QA^2^FDet requires 103.6 GFLOPs, lower than seven compared methods, and reduces the computational cost by 36.4% over QFDet while achieving higher accuracy. Although the highest FPS is not achieved, a favorable accuracy-complexity trade-off is obtained for UAV-based tiny pedestrian detection. This indicates that, when the object scale is extremely small and cross-modal misalignment is pronounced, relying solely on fusion strategies cannot effectively mitigate the performance limitations imposed by the inherent deficiencies of tiny-object representations. Similarly, confidence/evidence fusion methods (e.g., MCHE-CAF and CMPD) enhance fusion robustness through reliability modeling or multi-evidence aggregation but still exhibit limited improvements in tiny-object detection. Even when CMPD is scaled to 186.3 M parameters, its ${mAP}_{50}^{tiny}$ lags behind QA^2^FDet by 14.3%. These findings suggest that, in UAV tiny-object scenarios, the performance bottleneck is more likely attributed to insufficient fine-grained discriminability and misalignment-induced incorrect fusion or missed detections. Coarse-grained confidence weighting alone is often insufficient to resolve these issues.

We also evaluated several attention-based fusion detectors. Notably, transformer-based approaches did not demonstrate inherent advantages on RGBTDronePerson. For instance, C^2^Former, despite its strong attention modeling capability, underperforms QA^2^FDet by 8.9% in ${mAP}_{50}^{all}$. ICAFusion achieves competitive overall performance with higher throughput (39.6 FPS), but it is still surpassed by QA^2^FDet in both overall best-case performance (with an approximate relative gain of 4.4%) and in the extremely tiny subset, where ${mAP}_{50}^{tiny1}$ decreases by 6.3%. These results indicate that, in tiny-object scenarios, global modeling does not necessarily translate into consistent improvements in fine-grained discrimination or localization. In contrast, QFDet, a detector specifically designed for tiny UAV pedestrians, remains a strong baseline, achieving the best performance on 2 out of 7 metrics. QA^2^FDet trails by 7.1%/9.7% on ${mAP}_{50}^{tiny2}$/${mAP}_{50}^{small}$, respectively. This stage-wise discrepancy is likely attributable to the scale characteristics and sample distribution variability in the small and mid-tiny ranges, suggesting that further improvements could be obtained through enhanced scale coverage and mid-frequency shape modeling. Overall, the proposed method demonstrates more stable gains on key metrics. Compared with QFDet, QA^2^FDet achieves improvements of 7.1%/6.4%/3.6% in ${mAP}_{50}^{all}$/${mAP}_{50}^{tiny}$/${mAP}_{25}^{all}$, respectively, validating its effectiveness and robustness for UAV top-down RGBT tiny pedestrian detection.

**Figure 4:**
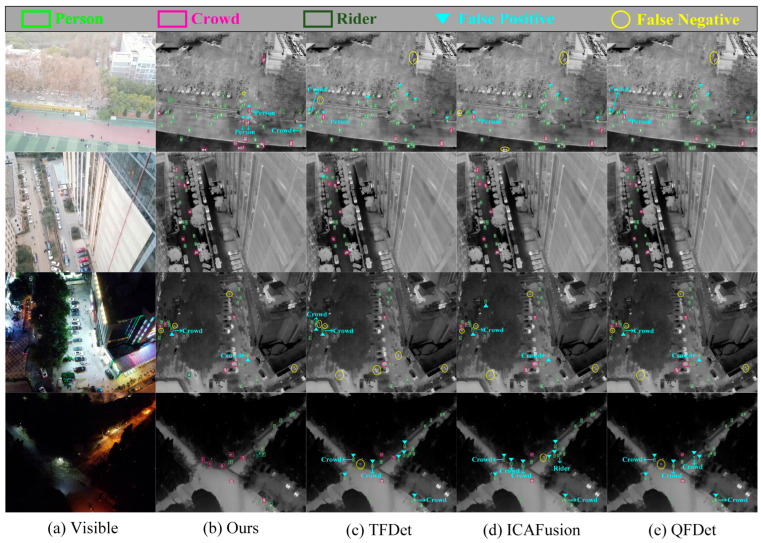
Qualitative comparison of detection results on the RGBTDronePerson dataset. Green, magenta, and gray boxes indicate the detected categories of Person, Crowd, and Rider, respectively. Cyan triangles and yellow circles indicate false positives and false negatives, respectively.

(2) Qualitative Comparison and Analysis: The qualitative comparisons among TFDet, ICAFusion, QFDet, and the proposed method are presented in Figure 4. The first column shows the original visible images, and the remaining columns present the detection results of different methods. Overall, our method achieves competitive or superior performance across diverse scenes, with fewer false positives (FPs) and false negatives (FNs) in more challenging cases. In Scene 1, where tiny objects are distributed over cluttered backgrounds, our method produces more complete detections while suppressing background-induced false alarms more effectively than the compared methods. This improvement is mainly associated with SDE, by which weak contours and fine details of tiny objects are better preserved. In Scene 2, all methods except TFDet achieve nearly error-free detections, indicating that this scene is relatively less challenging; in this case, the proposed method performs comparably to ICAFusion and QFDet, while avoiding the extra false alarm observed in TFDet. The stable predictions in this scene are consistent with the role of CCM in maintaining cross-modal correspondence. In Scenes 3 and 4, captured under nighttime or extremely low-illumination conditions, the advantage of our method becomes more evident. Under such conditions, QPE reduces the influence of degraded visible features by estimating modality reliability, while PGF adaptively adjusts feature fusion according to modality quality, leading to cleaner outputs and fewer missed detections. Nevertheless, occasional missed detections remain in extremely dark and heavily occluded regions, suggesting that severe object overlap and simultaneous degradation in both modalities continue to limit detection performance.

#### 3.2.2. Comparison on VTUAV-Det

**Figure 5:**
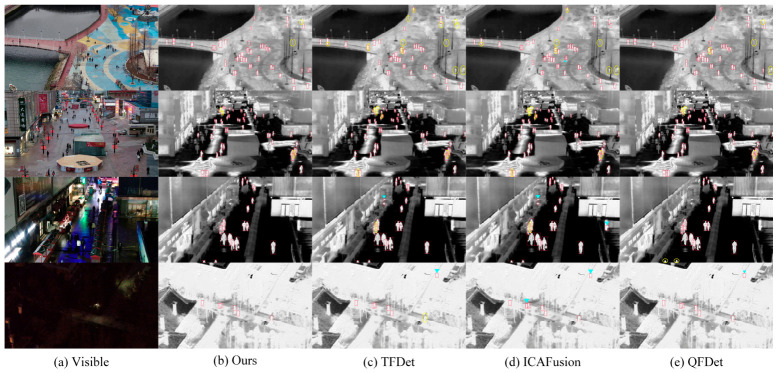
Qualitative comparison of detection results on the VTUAV-Det dataset.

(1) Quantitative Comparison and Analysis: On this dataset (Table 2), where object-scale variation is more pronounced, performance was evaluated using standard COCO metrics. Compared with the strongest baselines (CAGTDet and QFDet), our method yields gains of approximately 2.8% in mAP, 3.7% in mAP_50_, and 3.4% in the more stringent mAP_75_, indicating more consistent detection and localization accuracy. Scale-wise results further suggest improved robustness to scale variation: relative to CAGTDet, mAP_s_ increases by approximately 9.5% for small objects, and mAP_m_ improves by 6.9% for medium objects, which is plausibly attributed to the effectiveness of the proposed intra-modality detail-semantic joint enhancement design. Meanwhile, only a marginal gap is observed for large objects, with mAP_l_ being merely 0.18% lower than QFDet, suggesting that representation of extremely large objects could still be refined. In terms of efficiency, our method shows moderate computational complexity, requiring 101.7 GFLOPs, which is lower than that of seven compared methods. Compared with QFDet, the computational cost is reduced by approximately 36.6%, while superior overall detection performance is achieved. However, its inference speed is 55.9% lower than that of the fastest method, ICAFusion, and its parameter count is higher than those of lightweight SOTA detectors such as CAGTDet and TFDet. Therefore, although a favorable accuracy-complexity trade-off is obtained, the accuracy–efficiency balance remains to be further optimized.

(2) Qualitative Comparison and Analysis: Figure 5 shows the qualitative comparison results on the VTUAV-Det dataset. Four representative scenes with different object distributions and illumination conditions are selected to evaluate detection performance in complex environments. In Scene 1, small objects are unevenly distributed and locally crowded on the bridge and riverside, which increases the difficulty of discrimination under cluttered backgrounds. Compared with the competing methods, our method better separates adjacent objects and yields fewer missed detections. In Scene 2, objects appear in a complex urban road scene with strong background interference, occlusion, and scale variation. Under these conditions, weak small objects are more likely to be missed by the competing methods, whereas our method maintains higher sensitivity, resulting in fewer FNs. In Scene 3, two heavily occluded pedestrians are located in the lower part of the image, with only their heads visible. Owing to the extremely limited visual cues, this case is particularly challenging. Only our method and TFDet detect both objects, while ICAFusion and QFDet miss them. In addition, FPs are produced by TFDet and ICAFusion in background regions. By contrast, our method shows stronger robustness to severe occlusion and better suppression of background interference. In Scene 4, the visible image is severely degraded under extremely low illumination, making detection more dependent on thermal cues. Even in this case, the main objects are still reliably detected by our method. Nevertheless, some missed detections remain for ultra-small, heavily occluded, or weak-thermal objects, indicating that further improvement is needed in weak-object representation and dense small-object discrimination.

#### 3.2.3. Comparison on VTSaR

**Figure 6:**
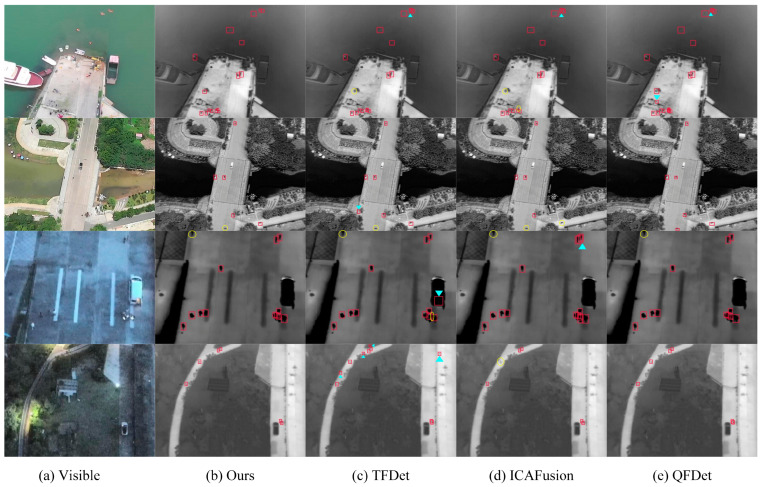
Qualitative comparison of detection results on the VTSaR dataset.

(1) Quantitative Comparison and Analysis: Based on Table 3, we compare our method with various methods on the VTSaR dataset, with particular attention to performance on different object scales. Our method improves overall accuracy by 1.60 mAP over C^2^Former and 1.20 mAP_50_ over QFDet. Meanwhile, under stricter localization requirements, mAP_75_ lags 0.70 behind the best method (QFDet) but outperforms the next-best method (TFDet) by 2.50, suggesting competitive high-IoU localization quality but not yet at the top level. The gain is mainly driven by small-object detection: mAP_s_ increase by 2.90 compared to the best small-object baseline (DeformCAT), indicating a significant alleviation of the small-object bottleneck. For medium-scale objects, mAP_m_ lags 1.20 behind the best performer (MCHE-CAF) but outperforms the next tier (QFDet/TFDet) by 0.70, showing that the improvement in small-object accuracy does not come at the expense of medium-scale performance.

(2) Qualitative Comparison and Analysis: Figure 6 displays qualitative comparisons on the VTSaR dataset. In the first row, sparse and distant small objects are distributed across the dock area. Under this condition, all objects are correctly detected by our method. Most competing methods exhibit incomplete responses to low-saliency objects, whereas QFDet introduces one FP despite producing no FNs. In the second row, objects are distributed along the bridge and adjacent riverside, where structural clutter and scale variation increase the difficulty of localization. In this example, the most accurate result is obtained by QFDet, with no observable FPs or FNs, whereas one residual FN is still produced by our method. In the third row, the scene is degraded by low contrast, and the presence of a dominant vehicle causes interference to nearby small objects. Under this condition, some competing methods either generate false alarms around the large object or fail to separate adjacent instances, whereas more accurate localization is achieved by our method, and background-induced errors are more effectively suppressed. In the fourth row, objects are located near the image boundary under low-illumination conditions. Reliable detections are maintained by both our method and QFDet, while the remaining methods are more vulnerable to boundary ambiguity and background clutter. Overall, our method demonstrates competitive performance on VTSaR, particularly in sparse and low-contrast small-object scenarios. However, this advantage is not consistent across all scenarios, and occasional errors are still observed in scenes with complex geometric structures. These observations suggest that further improvement is needed in fine-grained discrimination and prediction consistency for small objects in cluttered environments.

### 3.3. Ablation Studies and Discussion

#### 3.3.1. Overall Ablation

The results in Table 4 show that the proposed detail enhancement, cross-modal alignment, quality modeling, and the fusion strategy contribute materially to the overall performance. Relative to the Baseline, which uses a dual-stream backbone with naive modality concatenation, the full QA^2^FDet achieves an approximately 14.1% relative gain in ${mAP}_{50}^{all}$. Stepwise ablations further isolate the effect of each module. With SDE introduced (V1), ${mAP}_{50}^{all}$ is improved by about 3.9% over the Baseline, indicating that explicit high-frequency detail enhancement coupled with semantic gating increases the discriminability of shallow features. When CCM is further incorporated (V2), an additional 1.8% relative gain is obtained beyond V1, suggesting that thermal-guided local cross-window alignment is necessary and effective for alleviating cross-modal misalignment and exploiting complementary cues. After adding QPE (V3), although it is used only as an auxiliary supervised branch, performance is further improved by approximately 2.5% relative to the configuration without QPE, implying that explicit intra-modality detection-quality modeling facilitates learning more stable and robust representations. Within PGF, the contributions of the two information sources, namely the quality prior and modality discrepancy, were then examined. When only the quality prior weight W^Q^ is applied without the discrepancy weight W^D^ (V4), a further 1.1% gain over V3 is observed, indicating that the quality map can suppress interference from low-quality regions to a certain extent. When only W^D^ is retained (V5), a substantially larger improvement of approximately 3.8% over V3 is achieved, and performance remains clearly higher than the quality-only variant, with about 2.7% over V4. This confirms that discrepancy-aware gated residuals are more critical for characterizing cross-modal complementarity and emphasizing the more reliable modality. Finally, when W^Q^ and W^D^ are jointly incorporated into PGF, performance is further improved by approximately 1.4% over the discrepancy-only setting, highlighting the importance of the quality prior in guiding cross-modal reliability trade-offs and suppressing degraded modalities.

Model complexity was further evaluated in terms of parameter count, inference throughput, and GFLOPs. As the proposed modules are progressively integrated, GFLOPs increase from 78.8 in the baseline to 103.6 in the full model, while ${mAP}_{50}^{all}$ improves from 39.56% to 45.13%. This suggests that the increased computational cost is effectively converted into detection accuracy. Notably, QPE incurs only a marginal computational increase, whereas PGF provides further performance gains with additional overhead. Overall, the results demonstrate the complementarity and synergy among the proposed modules, achieving a favorable accuracy-complexity trade-off for robust RGBT pedestrian detection in tiny-object and complex-scene scenarios.

Although QA^2^FDet improves detection accuracy and robustness, its computational cost should be considered for real-time UAV deployment. As reported in Table 1, Table 2, Table 3 and Table 4, QA^2^FDet is designed as an accuracy- and robustness-oriented detector rather than an ultra-lightweight model, aiming to jointly address weak tiny-object representation, cross-modal misalignment, and spatially varying modality reliability. The module-wise ablation results indicate that the main computational overhead comes from SDE, CCM, and PGF. SDE increases the parameter count by introducing additional feature-enhancement branches for fine-grained tiny-object cues. CCM is a major source of latency because it involves window partitioning, token rearrangement, and local cross-modal attention, whose runtime cost is not fully reflected by GFLOPs. PGF further introduces extra operations for quality- and discrepancy-aware gated fusion, whereas QPE remains lightweight and adds only marginal overhead. Therefore, CCM and PGF are the primary computational bottlenecks of the current framework.

For embedded UAV deployment, QA^2^FDet is more suitable for platforms with moderate onboard computing capability than for highly resource-constrained micro-UAV processors. The reported throughput can support offline analysis or low-frame-rate onboard perception, while strict real-time deployment on power-limited embedded devices may require further optimization. Potential directions include replacing ResNet-50 with a lightweight backbone, enlarging the CCM window size to reduce local matching cost, and using hardware-specific inference engines. Since this work focuses primarily on robust RGBT tiny pedestrian detection under misalignment and modality-quality fluctuation, embedded power profiling and hardware-aware model compression are left for future work.

#### 3.3.2. Ablation on SDE

As shown in Table 5, a systematic ablation was conducted on the three key components of SDE, namely Block-DCT-based high-frequency enhancement, semantic gating, and the refinement module. When only the Block-DCT branch was enabled, performance was improved by approximately 1.31% over the baseline, indicating that explicit high-frequency enhancement strengthens the perception of small object contours and fine-grained structures. After semantic gating was further introduced, an additional gain of approximately 1.60% was obtained, suggesting that semantic selection within high-frequency features, by emphasizing detection-relevant responses while suppressing background noise, is critical for improving feature discriminability. With the refinement module incorporated, which is implemented using attention and depthwise separable convolution, performance was further increased by approximately 0.91% relative to the semantic gating variant. Although the gain is modest, it indicates that fine calibration of fused features and local context modeling remains beneficial in complex backgrounds. Overall, the complete SDE achieves a cumulative improvement of approximately 3.87% over the baseline, with an 11.2% increase in GFLOPs, a 51.4% increase in parameter count, and a 10.6% decrease in inference throughput. This suggests that the complete SDE improves detection performance without incurring excessive computational burden.

#### 3.3.3. Sensitivity Analysis of the CCM Window Size

As seen in Table 6, b = 4 achieves the highest ${mAP}_{50}^{all}$ with a moderate computational overhead. When b = 2, the number of local windows is increased, resulting in higher GFLOPs and lower FPS. In contrast, larger window sizes, such as b = 8 and b = 16, reduce the computational cost but lead to inferior detection accuracy, as excessive background information is introduced into each window and fine-grained cross-modal correspondence is weakened. Therefore, b = 4 is adopted as the default window size in CCM.

#### 3.3.4. Robustness to Cross-Modal Misalignment and Temporal Asynchrony

To further assess the robustness of QA^2^FDet to cross-modal misalignment, both controlled synthetic perturbation experiments and practical temporal-asynchrony evaluations are conducted. The former are used as controlled stress tests, whereas the latter are designed to emulate synchronization errors commonly encountered in UAV-based RGBT acquisition.

**Figure 7:**
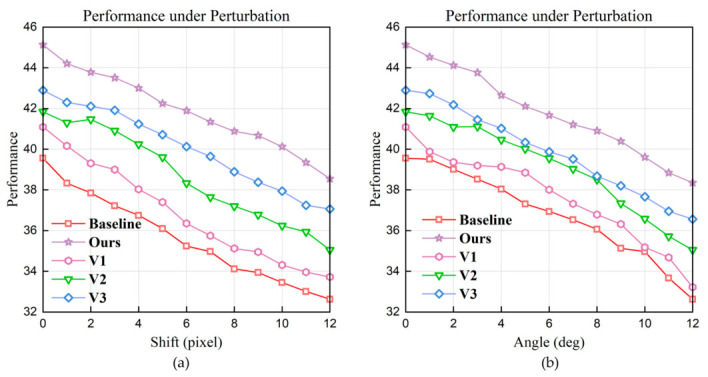
Robustness comparison of five models under synthetic geometric perturbations. (**a**) Performance under incremental angular rotations; (**b**) Performance under incremental pixel-wise shifts.

For the controlled stress test, two types of synthetic geometric perturbations were introduced: center rotation with progressively increasing angles and translation bias with progressively increasing pixel offsets (corresponding to Figure 7a,b). The same perturbations were applied to the baseline, the ablation variants (V1–V3), and the full model for a fair comparison. As the perturbation magnitude increased, performance consistently degraded for all methods. Nevertheless, across the full ranges of both perturbation types, QA^2^FDet achieved the best performance and exhibited a noticeably slower degradation trend, indicating stronger tolerance to misalignment. This behavior is consistent with the design motivation: small object detection is highly sensitive to cross-modal misalignment. By leveraging CCM to mine thermal-guided, fine-grained cross-modal correspondences and perform local neighborhood alignment, the risk of feature drift induced by geometric deviations was effectively reduced.

Although rotation and translation perturbations can reveal the sensitivity of different models to controlled spatial deviations, they do not fully reflect practical UAV-RGBT acquisition errors. In real UAV systems, visible and thermal sensors may suffer from imperfect temporal synchronization due to differences in sensor exposure, transmission latency, frame buffering, or onboard processing pipelines. VTUAV-det is selected for this temporal-asynchrony evaluation because it is re-annotated from UAV tracking sequences and preserves the temporal order of visible–thermal frames, making it suitable for constructing delayed RGBT pairs. Therefore, we further construct temporally asynchronous RGBT pairs on VTUAV-det to simulate practical UAV-induced synchronization errors.

As shown in Table 7, temporal asynchrony consistently increases the relative mAP_50_ degradation of all variants, indicating that UAV-RGBT detection is sensitive to inter-modal synchronization errors. The baseline shows a severe relative degradation of 16.59% at |Δ| = 5. With the progressive introduction of SDE, CCM, and QPE, this degradation is gradually mitigated, demonstrating that tiny-object feature enhancement, local cross-modal correspondence modeling, and modality-quality estimation improve robustness to delayed RGBT inputs.

QA^2^FDet achieves the lowest relative mAP_50_ degradation under all delay settings. At |Δ| = 5, its relative degradation is only 6.93%, corresponding to a 58.23% reduction compared with the baseline. These results demonstrate that the proposed quality-aware adaptive alignment-fusion strategy effectively enhances robustness against temporal synchronization errors, especially under large inter-modal delays.

#### 3.3.5. Intermediate Feature Visualization and Interpretability Analysis

**Figure 8:**
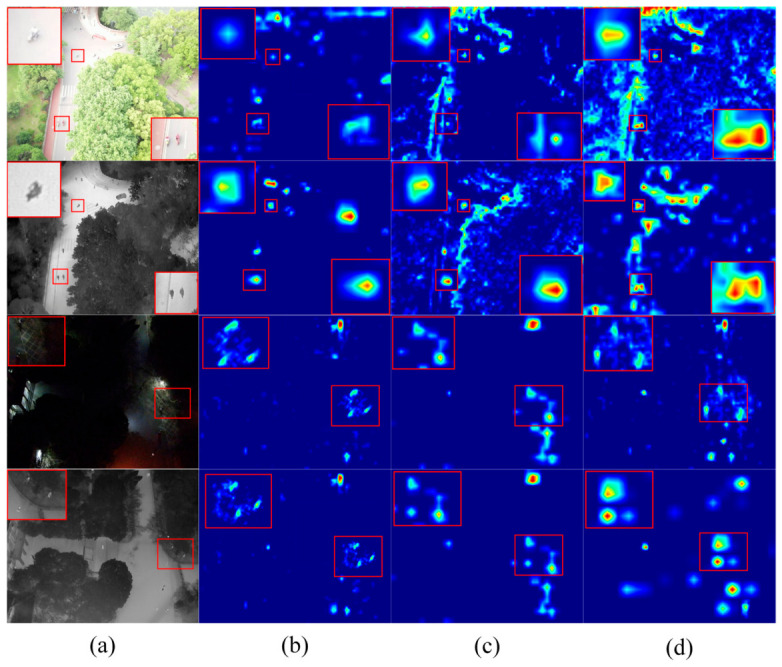
Heatmap visualization of feature responses at different stages on RGBTDronePerson. For each image pair, the upper row corresponds to the visible modality, and the lower row corresponds to the infrared modality. The red boxes denote the object regions, where the corresponding object features are magnified to facilitate visual comparison. From left to right, the columns show (**a**) the input images, (**b**) backbone features, (**c**) SDE enhanced features, and (**d**) modality-specific features before cross-modal fusion, respectively.

As shown in Figure 8, feature responses at different stages of the network are visualized to assess the progressive effect of the proposed modules. Although the backbone features can roughly localize the object regions, their activations remain diffuse, and noticeable non-target responses are observed in cluttered backgrounds, edge structures, and low-illumination areas, indicating limited discriminability of the original representations. After refinement by the SDE module, activations over the target regions become more compact, while background clutter and irrelevant responses are significantly suppressed. This effect is particularly evident for small and low-saliency targets, whose response completeness and contrast are both improved, suggesting that the SDE module effectively strengthens intra-modal fine-grained representations and enhances object saliency. Before the final fusion stage, the features are further processed by the CCM, where cross-modal correspondences are explicitly modeled. As a result, more consistent and stable object-oriented activation patterns are obtained across the two modalities, while modality-specific irrelevant responses are further attenuated. These observations indicate that the CCM effectively improves cross-modal association modeling and feature alignment, thereby providing cleaner and more discriminative inputs for subsequent fusion and ultimately enhancing the overall detection performance.

To complement the qualitative observations, we conduct a quantitative interpretability analysis on the validation set. The effect of SDE is examined from both frequency and spatial perspectives: the low-frequency energy ratio is obtained by applying the same 8 × 8 Block-DCT to feature maps before and after enhancement, while the foreground-to-background response contrast is calculated from channel-averaged activation maps using ground-truth boxes as foreground masks. For QPE, we measure the Pearson correlation between the predicted quality-prior maps and the IoU-based target quality maps. The foreground-to-background quality ratio is also reported to examine whether QPE assigns higher reliability scores to object regions. To analyze PGF, we evaluate gate-quality consistency, which indicates whether the learned modality gates are consistent with the relative modality quality estimated by QPE. Finally, the effectiveness of CCM is assessed by comparing the cosine similarity between visible and thermal features within ground-truth regions before and after correspondence mining.

Table 8 provides quantitative evidence that is consistent with the visual observations in Figure 8. After SDE, the low-frequency energy ratio decreases by 0.133, while the foreground-to-background response contrast increases by 0.550. These results indicate that SDE suppresses redundant low-frequency background responses and enhances object-related activations, which agrees with the clearer and more concentrated pedestrian responses shown in Figure 8. For CCM, the cross-modal similarity within ground-truth regions improves by 0.134, confirming that correspondence mining promotes better visible–thermal feature alignment. This is also reflected in Figure 8, where the cross-modal responses become more consistent after alignment.

For QPE and PGF, since the reference model does not contain the corresponding branches, we analyze their intrinsic reliability. The predicted quality-prior maps achieve positive correlations of 0.681 and 0.714 with the IoU-based target maps for the visible and thermal modalities, respectively. Moreover, the foreground-to-background quality ratios of 2.170 and 2.450 show that QPE assigns higher reliability scores to object regions. PGF further obtains a gate-quality consistency of 76.8%, indicating that its learned gates largely follow the modality reliability estimated by QPE.

## 4. Conclusions

UAV-based RGBT tiny pedestrian detection is challenging due to the limited discriminability of tiny objects in aerial images and the difficulty of achieving reliable multimodal fusion under complex backgrounds and inconsistent modality quality. To this end, we propose a quality-aware adaptive alignment fusion network, termed QA^2^FDet. The proposed method improves tiny pedestrian detection by enhancing discriminative target representation, promoting more reliable cross-modal interaction, and enabling adaptive fusion according to regional modality quality, thereby yielding more robust performance in complex aerial environments. Extensive experiments on multiple UAV-based RGBT detection benchmarks demonstrate that our method achieves competitive performance and improves detection accuracy in challenging scenarios with complex backgrounds and illumination variations, validating the effectiveness of the proposed framework for UAV-based RGBT tiny pedestrian detection.

Despite these improvements, several limitations remain. In particular, the proposed framework may still encounter missed detections in extremely challenging situations, such as severe occlusion, very weak thermal responses, and highly cluttered structural backgrounds. Moreover, the enhanced performance is accompanied by additional computational overhead, especially from local correspondence mining and gated fusion, which may limit deployment efficiency on resource-constrained UAV platforms. Future work will focus on lightweight backbone replacement, structured pruning, quantization-aware training, and hardware-specific acceleration to improve real-time and energy-efficient deployment on embedded UAV systems.

## Figures and Tables

**Table 1:** Performance comparison with SOTA methods on RGBTDronePerson. “tiny1”, “tiny2”, “tiny3”, and “small” denote absolute object sizes that are in the range of [0, 8), [9, 12), [12, 20), and [20, 32], respectively. Red indicates the best result. Blue indicates the second-best result.

Method	${mAP}_{50}^{all}$	${mAP}_{50}^{tiny}$	${mAP}_{50}^{tiny1}$	${mAP}_{50}^{tiny2}$	${mAP}_{50}^{tiny3}$	${mAP}_{50}^{small}$	${mAP}_{25}^{all}$	FPS	Params	GFLOPs
(TMM’ 23) CMPD	38.56	40.42	18.75	31.86	49.66	24.93	55.83	10.7	186.3 M	130.2
(ISPRS’ 23) QFDet	42.15	44.32	20.33	37.62	50.87	28.60	58.63	20.9	63.4 M	162.9
(TMM’ 23) MCHE-CAF	29.20	30.76	15.38	24.73	33.59	26.63	43.71	22.8	78.9 M	115.4
(TIM’ 23) TINet	27.43	27.59	2.08	20.52	35.68	24.35	38.20	23.8	100.6 M	92.8
(TGRS’ 24) C^2^Former	41.08	43.36	17.39	30.89	48.35	25.02	57.26	20.3	133.0 M	145.5
(TITS’ 24) MS-DETR	42.32	43.09	19.88	31.92	48.75	24.87	56.45	15.6	151.2 M	163.5
(PR’ 24) CAGTDet	41.77	43.21	17.21	31.93	49.50	26.05	58.11	18.3	59.8 M	50.6
(IF’ 24) ICAFusion	43.22	44.06	20.74	31.87	50.34	26.17	57.91	39.6	125.7 M	147.2
(TNNLS’ 25) TFDet	33.58	36.28	15.94	29.53	43.86	28.13	49.56	14.5	53.7 M	98.9
(TMM’ 25) DeformCAT	40.34	42.68	19.58	33.57	51.87	24.61	56.92	25.7	120.6 M	158.9
Ours	45.13	47.16	22.13	34.96	53.35	25.83	60.74	16.9	89.3 M	103.6

**Table 2:** Performance comparison with SOTA methods on VTUAV-det. “s”, “m”, and “l” denote objects with an area in the range of are [0^2^, 32^2^), [32^2^, 96^2^), and [962, 1010], respectively. Red indicates the best result. Blue indicates the second-best result.

Method	mAP	mAP_50_	mAP_75_	mAP*_s_*	mAP*_m_*	mAP*_l_*	FPS	Params	GFLOPs
(TMM’ 23) CMPD	22.80	55.60	17.80	5.40	24.10	52.90	11.6	182.8 M	128.8
(ISPRS’ 23) QFDet	31.80	72.30	23.50	13.30	30.70	57.00	23.1	60.5 M	160.3
(TMM’ 23) MCHE-CAF	26.30	59.80	20.70	1.60	28.50	53.90	22.8	77.8 M	109.2
(TIM’ 23) TINet	27.20	60.80	20.20	2.00	29.40	51.80	24.0	100.1 M	91.2
(TGRS’ 24) C^2^Former	29.90	68.50	22.10	11.30	29.70	55.90	20.7	131.3 M	140.6
(TITS’ 24) MS-DETR	27.80	68.30	19.80	11.50	28.10	53.60	16.9	142.7 M	155.8
(PR’ 24) CAGTDet	32.10	71.60	22.50	15.80	30.30	56.10	18.3	59.5 M	49.8
(IF’ 24) ICAFusion	30.10	69.20	21.80	12.70	29.30	55.60	40.1	123.3 M	145.5
(TNNLS’ 25) TFDet	29.80	69.50	21.90	12.20	29.10	56.30	15.2	50.5 M	98.0
(TMM’ 25) DeformCAT	28.60	70.90	22.10	9.80	31.80	55.50	26.0	118.7 M	156.9
Ours	33.00	75.00	24.30	17.30	32.40	56.90	17.7	83.3 M	101.7

**Table 3:** Performance comparison with SOTA methods on VTSaR. “s” and “m” denote objects with an area in the range of are [0^2^, 32^2^) and [32^2^, 96^2^), respectively. Red indicates the best result. Blue indicates the second-best result.

Method	mAP	mAP_50_	mAP_75_	mAP*_s_*	mAP*_m_*	FPS	Params	GFLOPs
(TMM’ 23) CMPD	38.30	91.70	22.50	38.20	49.10	12.2	182.8 M	128.8
(ISPRS’ 23) QFDet	40.60	93.90	27.00	38.50	50.30	23.9	60.5 M	160.3
(TMM’ 23) MCHE-CAF	38.50	90.40	22.10	37.40	52.20	23.8	77.8 M	109.2
(TIM’ 23) TINet	37.20	90.10	21.90	36.30	47.30	25.2	100.1 M	91.2
(TGRS’ 24) C^2^Former	41.20	93.20	22.60	34.20	48.60	21.6	131.3 M	140.6
(TITS’ 24) MS-DETR	37.30	90.40	21.70	35.70	49.20	18.3	142.7 M	155.8
(PR’ 24) CAGTDet	39.30	92.20	23.50	38.60	48.90	19.0	59.5 M	49.8
(IF’ 24) ICAFusion	37.40	90.60	20.30	35.60	47.20	40.8	123.3 M	145.5
(TNNLS’ 25) TFDet	40.30	92.30	23.80	37.70	50.30	16.8	50.5 M	98.0
(TMM’ 25) DeformCAT	38.50	91.60	23.70	38.90	49.30	27.3	118.7 M	156.9
Ours	42.80	95.10	26.30	41.80	51.00	18.8	83.3 M	101.7

**Table 4:** Ablation study evaluating the effectiveness of the designed modules on RGBTDronePerson. “√” indicates that the corresponding module is incorporated in the variant, while a blank cell indicates that the module is not used. In the PGF column, “only *W^Q^*” and “only *W^D^*” denote that only the *W^Q^* branch or the *W^D^* branch of the PGF module is used, respectively. Red indicates the best result.

Variant	SDE	CCM	QPE	PGF	${mAP}_{50}^{all}$	FPS	Params	GFLOPs
Baseline					39.56	30.3	41.8 M	78.8
V1	√				41.09	27.1	63.3 M	87.6
V2	√	√			41.84	20.4	78.1 M	90.1
V3	√	√	√		42.89	19.3	78.8 M	90.7
V4	√	√	√	only *W^Q^*	43.37	19.0	80.7 M	92.5
V5	√	√	√	only *W^D^*	44.52	17.6	86.5 M	100.2
Ours	√	√	√	√	45.13	16.9	89.3 M	103.6

**Table 5:** Ablation study on the SDE module. Red indicates the best result. “√” indicates that the corresponding module is incorporated in the variant, while a blank cell indicates that the module is not used.

Variant	Block-DCT	Semantic Gating	Refinement	${mAP}_{50}^{all}$	FPS	Params	GFLOPs
Baseline				39.56	30.3	41.8 M	78.8
V6	√			40.08	29.6	48.1 M	82.3
V7	√	√		40.72	28.0	57.5 M	84.9
V1	√	√	√	41.09	27.1	63.3 M	87.6

**Table 6:** Sensitivity analysis of the CCM window size b. Red indicates the best result.

b	${mAP}_{50}^{all}$	FPS	Params	GFLOPs
2	44.38	14.6	89.3 M	116.2
4	45.13	16.9	89.3 M	103.6
8	44.15	18.1	89.3 M	96.8
16	42.07	18.8	89.3 M	93.4

**Table 7:** Robustness comparison under temporal synchronization errors on VTUAV-det. The results are reported in terms of mAP_50_. “Drop” denotes the absolute decrease from the synchronized setting Δ = 0, with +Δ and −Δ averaged for each |Δ|.

Variant	Δ = 0	|Δ| = 1	Drop	|Δ| = 3	Drop	|Δ| = 5	Drop
Baseline	63.30	60.70	2.60	56.90	6.40	52.80	10.50
V1	66.51	64.15	2.36	61.10	5.41	57.68	8.83
V2	68.08	66.30	1.78	63.65	4.43	60.95	7.13
V3	70.29	68.65	1.64	66.55	3.74	64.15	6.14
Ours	75.00	73.80	1.20	72.05	2.95	69.80	5.20

**Table 8:** Interpretability analysis of the proposed modules. $f_{m}$ is the input feature of modality *m*, ${\tilde{f}}_{m}$ is the SDE-enhanced feature, $f_{V}^{a}$ is the CCM-aligned visible feature, $Q_{m}$ and $Q_{m}^{*}$ are the QPE-predicted and IoU-based target quality maps, and *W_m_* is the final PGF gate. Newly introduced statistics are $R_{LF}$, $C_{fg/bg}$, $S_{cm}$, $R_{Q}$, and sgn, denoting low-frequency energy ratio, foreground-background response contrast, cross-modal cosine similarity in GT regions, foreground-background quality ratio, and the sign function used to measure the directional consistency between modality-quality differences and PGF-gate differences, respectively.

Module	Metric	Reference	QA^2^FDet
SDE	$R_{LF}{(f}_{m})>R_{LF}({\tilde{f}}_{m})$	0.642	0.509
SDE	$C_{fg/bg}{(f}_{m})<C_{fg/bg}({\tilde{f}}_{m})$	1.360	1.910
CCM	$S_{cm}(f_{V},f_{T})<S_{cm}(f_{V}^{a},f_{T})$	0.438	0.572
QPE	$Corr(Q_{m},Q_{m}^{*})$ (V/T)	-	0.681/0.714
QPE	$R_{Q}(Q_{m})$ (V/T)	-	2.170/2.450
PGF	$sgn\left(Q_{T}-Q_{V}\right)=sgn(W_{T}-W_{V})$	-	76.8%

## Data Availability

Data are contained within this article.
